# Bone Marrow Cells in Murine Colitis: Multi-Signal Analysis Confirms Pericryptal Myofibroblast Engraftment without Epithelial Involvement

**DOI:** 10.1371/journal.pone.0026082

**Published:** 2011-10-13

**Authors:** Chung-Yin Lee, Rosemary Jeffery, Gillian Hutchinson, Malcolm R. Alison, Richard Poulsom, Nicholas A. Wright, William R. Otto

**Affiliations:** 1 Histopathology Laboratory, Cancer Research UK, London Research Institute, London, United Kingdom; 2 Biological Resources Unit, Cancer Research UK, London Research Institute, London, United Kingdom; 3 Centre for Digestive Diseases, Blizard Institute, Barts and The London School of Medicine and Dentistry, London, United Kingdom; University Hospital Freiburg, Germany

## Abstract

**Background:**

The contribution of bone marrow-derived cells to epithelial tissues in the inflamed gut remains controversial. Recent reports have suggested that cell fusion between bone marrow-derived cells and the intestinal epithelium takes place in inflammatory conditions.

**Methods:**

In attempts to confirm this, we have undertaken gender mis-matched bone marrow (BM) transplants from male Swiss Webster (SWR) mice to B and T cell-deficient female Rag2 KO mice which, 4 weeks later, were given 5% dextran sodium sulphate in drinking water to induce acute colitis. A further BM-treated group of animals with a graft versus host-like condition was also studied. We developed a new method to combine up to three brightfield or fluorescent lectin- or immuno-histochemical signals with fluorescent *in situ* hybridisation for the Y and X chromosomes to enable us unequivocally to identify BM-derived male cells which presented as different cell types in the gastrointestinal tract.

**Principal Findings:**

In rolled preparations of whole intestines we scanned around 1.5 million crypts at many tissue levels. In no instance did we see a Y chromosome-positive cell in the epithelial compartment, which was not also CD45-positive. We saw no evidence of cell fusion, based on combined X and Y chromosome analysis. Levels of CD45-positive stromal and lymphoid cells and pericryptal myfibroblasts (positive for α-smooth muscle actin) increased with time up to a plateau, which resembled the level seen in untreated control grafted animals. We saw very few Y chromosome-positive endothelial cells in intestinal stromal vessels.

**Conclusions:**

We conclude that whole BM transplantation does not result in intestinal epithelial engraftment in this model. Our new methods can usefully assist in multi-signal analyses of cell phenotypes following BM transplant and in models of chimaerism and regenerative medicine.

## Introduction

Several laboratories, including our own [1]–[7], have described the phenomenon of bone marrow (BM)-derived cells engrafting into organs such as the liver, kidney, skin, blood vessels as well as tumour stroma [8]–[13]. Such studies commonly used an isogenic male-into-female bone marrow reconstitution after lethal irradiation, and engraftment was followed using analysis of Y-chromosome-specific DNA probes by *in situ* hybridisation (ISH), whether chromogenic or fluorescent (FISH). Studies of the kidney [14] and liver [8] have demonstrated that a small proportion of putative engraftment events were in fact cell fusions between parenchymal cells and those from the bone marrow. There have been reports of engraftment of BM cells into the three main differentiated cell types of the human intestinal epithelium (mucous or goblet cells, absorptive and enteroendocrine cells), but these have only been seen as individual cells in areas of inflammation, and not as clonal, whole-crypt stem cell events [4]–[6]. More recently, Davies and colleagues [15] have reported cell fusion as a major mode of bone marrow engraftment into the intestinal tissues of mice with experimental inflammation, in studies which suggested whole crypt involvement. In addition, Wei and co-workers [16] infused cultured and bromo-deoxyuridine (BrdU)-prelabelled male haematopoietic and mesenchymal stem cells from bone marrow into female rats that had TNBS colitis. They found both BrdU and Y chromosome label in large contiguous areas of the colon, including whole crypts, at 21 days after infusion. These widely differing reports demonstrate the continuing controversy about the ability and extent to which the bone marrow may contribute to epithelial tissues of the gut, and suggest that this is still a matter worthy of re-examination.

We have sought to confirm whether bone marrow cells can engraft into the gastrointestinal tract by taking advantage of the *Dolichos biflorus* agglutinin (DBA; from Indian horse gram seed) reverse chromophilia in different mouse strains [17]. C57 Black 6J (C57BL/6J) mice display a DBA lectin profile in their intestinal tissue of positivity in the epithelium, whereas their blood vessel endothelial cells are DBA-negative. In contrast, Swiss Webster (SWR) mice show a reverse pattern. These strains have been used to investigate gastrointestinal (GI) clonality in blastocyst chimaeras, where crypt epithelial cells of the small and large bowel are either positive or negative for the lectin, indicating the likelihood of a clonal crypt architecture [18]–[20]. We hypothesised that after transplantation of male SWR bone marrow (BM) into lethally-irradiated female recombination activating gene-2 (Rag-2) mice (on a C57BL/6J background) there would be a likelihood that reversal of lectin staining would indicate the engraftment of BM-derived cells into either the epithelium or blood vessels. Rag-2 mice have a deficiency in T and B cells as a result of that enzyme loss [21]. Such mice were engrafted and then challenged with dextran sodium sulphate (DSS) to induce an acute colitis. We have thus sought to augment any naturally-occurring low level engraftment dynamics by creating an inflammatory milieu specifically within the colon. This approach has been used many times in intestinal biology [22]–[24]. Chronic inflammation has also been induced in the stomach in experimental gastric carcinoma induction by *Helicobacter felis* infection, where bone-marrow-derived cells were reported to take part in tumour formation [25]. However, in many studies of engraftment by BM-derived cells it is often unclear which donor cell phenotypes have been detected in the tissues under study. We have overcome this by analysing intestinal tissues using combinations of dual or triple brightfield and fluorescent overlay lectin- or immunohistochemistry combined with fluorescent X and Y chromosome *in situ* hybridisation (FISH) on the same tissue section. By doing this we hoped to ensure that any cells of bone marrow origin that were detected were unequivocally of specific phenotypes, with additional indications of the presence or absence of cell-cell fusion. We found that all of the donor-derived Y-chromosome-positive cells seen within the epithelial compartment were CD45-positive, and thus of lymphoid origin, and so we found no gut epithelial cells which could be said to have differentiated from the bone marrow.

## Results

### DBA Lectin Histochemistry

**Figure 1:**
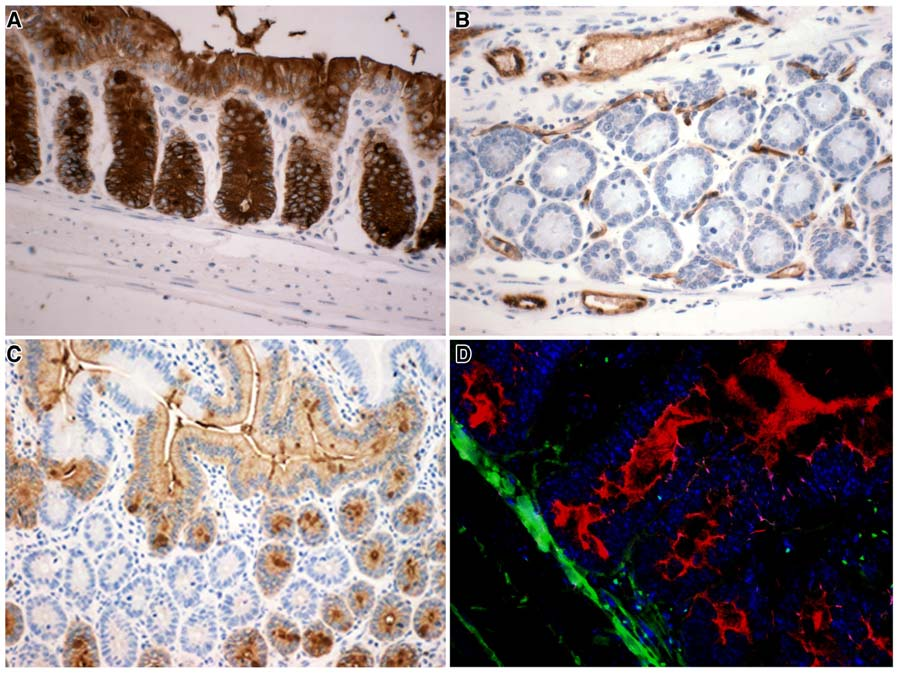
Lectin histochemistry. *Dolichos biflorus* agglutinin histochemistry of intestinal epithelia revealed by peroxidase-conjugated DBA and DAB in (A) C57 black 6J colon, (B) SWR colon, (C) Small intestine from an aggregation chimaera between both mouse strains. (D) C57 Black 6J small intestine revealed by alkaline phosphatase-conjugated DBA using Vector Red with DAPI nuclear stain in blue subsequently probed for Y chromosomes (green dots) to give 3 signals on the section.

The *Dolichos biflorus* agglutinin brightfield lectin histochemistry gave good histological signals (Fig 1). As expected Rag-2 KO mice (C57BL/6J background; Fig 1A) had DBA-positive epithelium and negative endothelium, whereas the SWR mice had the reverse phenotype (Fig 1B). An aggregation chimaera between both strains of mice showed the ability to distinguish clearly individual cells of each genotype, where crypts are clonal, but villi may be fed by both genotypes (Fig 1C). The C57BL/6J background phenotype was also revealed to be similar to the Rag-2 KO using an AP-lectin conjugate with the substrate Vector Red in fluorescent mode. The section was then probed for male cells using the Y chromosome paint (Fig 1D, green nuclear dots, with DAPI nuclear stain). No attempt to suppress autofluorescence was undertaken since we found that this usefully defined aspects of tissue morphology.

### IHC

**Figure 2:**
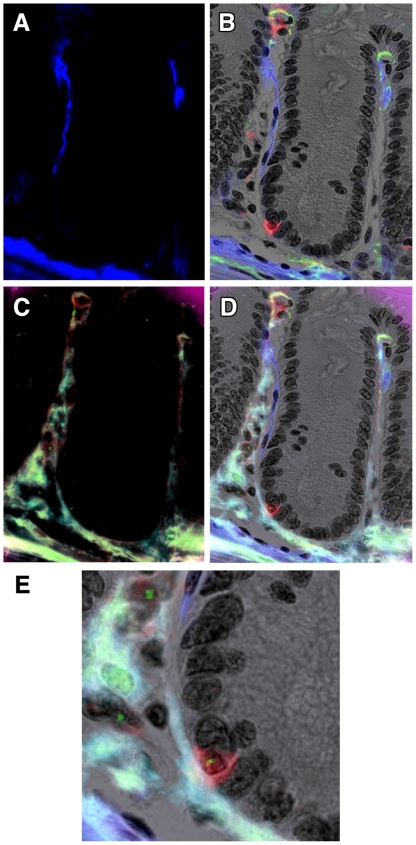
Double IHC for α-SMA and CD45 with Y chromosome paint. A colonic crypt from a female Rag2^−/−^ mouse transplanted 28 days earlier with male SWR BM. A. α-SMA was stained using two layer IHC with a Cy5-conjugated second layer, here false-coloured blue. B. CD45 was revealed using 3 layer IHC using Vector Red as chromogen for alkaline phosphatase-conjugated streptavidin, here revealed in fluorescent mode using the Cy3 filter, in combination with the brightfield greyscale image (with nuclei stained with haematoxylin), and overlaid with the α-SMA image from A. Note the close morphology of the CD45-positive cell to that of its neighbours. C. Subsequent Y chromosome paint (green nuclear spots, FITC filter) on the same section. Note background fluorescence revealing some tissue morphology. D. Overlay image of B and C showing the CD45 cell to be Y chromosome-positive, together with some stromal male cells. E. Magnified view of part of D. Original magnifications: A-D 200X, E 400X.

**Figure 3:**
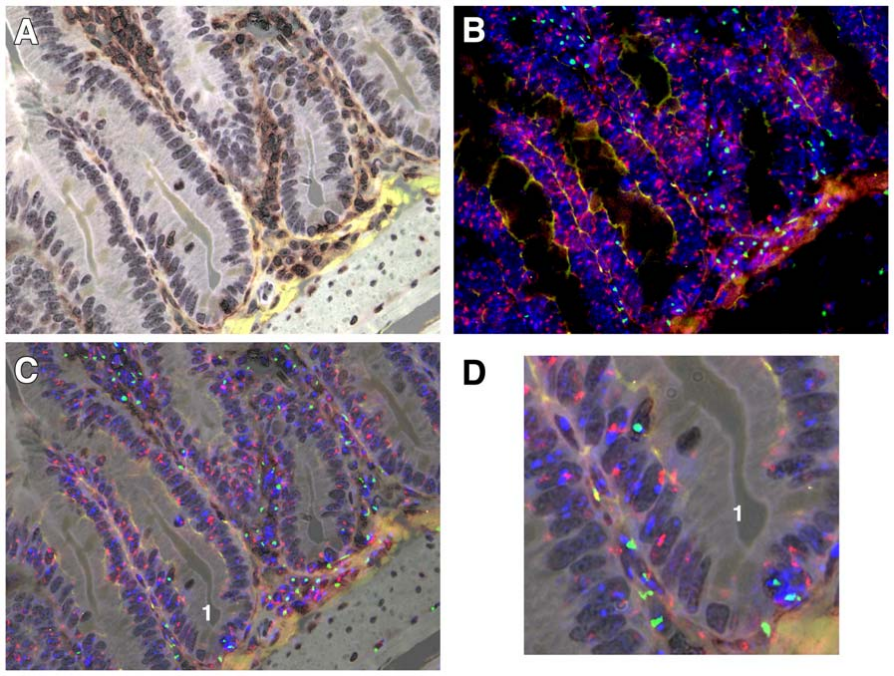
Combined CD45 IHC with XY chromosome paints. A. Female Rag2^−/^ small intestine stained with 3 layer IHC for CD45 and revealed with DAB (brown), with light haematoxylin nuclear counterstain. B. Same section probed sequentially for X (red) and Y (green) chromosomes, with nuclei additionally stained with DAPI (blue). Note background fluorescence (yellow-orange) yielding some tissue morphology. C. Overlay image of A and B revealing crypts containing several possible male epithelial cells (No 1). D. Enlarged image of portion of C. Note male cells apposed to crypt base, and also within the epithelial layer, the latter being CD45 positive. A–C 200X, D digitally enlarged to 400X.

The CD45 antibody reliably stained a large proportion of cells in Peyer's patches, a useful internal control for monitoring bone marrow engraftment, as well as infiltrating lymphocytes within the epithelial compartment, some of which looked remarkably like epithelial cells. The antibody was useful in both fluorescent and brightfield modes (Figs 2 and 3 respectively), and stained sections could be subsequently reprobed with Y and/or X chromosome paints to reveal the genotypes of particular cells of interest. In combination with α-SMA IHC (Fig 2A), such analyses revealed occasional apparently epithelial cells in the colon, but which were always positive for CD45 (Fig 2B), and always found to be Y chromosome positive (Fig 2C, D, E).

The CD45 antibody revealed areas of inflammation between crypts in female mice displaying a GvHD-like condition 28 days after BMTx (Fig 3). There were many stromal CD45-positive cells and also some within the epithelial compartment (Fig 3A, brown stained cells). Subsequent XY chromosome analysis revealed many male bone marrow-derived cells (Fig 3B). By combining these images it became clear that a Y chromosome-positive, apparently epithelial cell, in one crypt was also CD45-positive (Fig 3C), which is shown at higher magnification in Fig 3D. We saw no evidence of cells with higher XY ratios, suggesting that cell fusion was not a factor. Of interest were some male cells within the muscularis mucosae beneath the crypts (Fig 3C).

**Figure 4:**
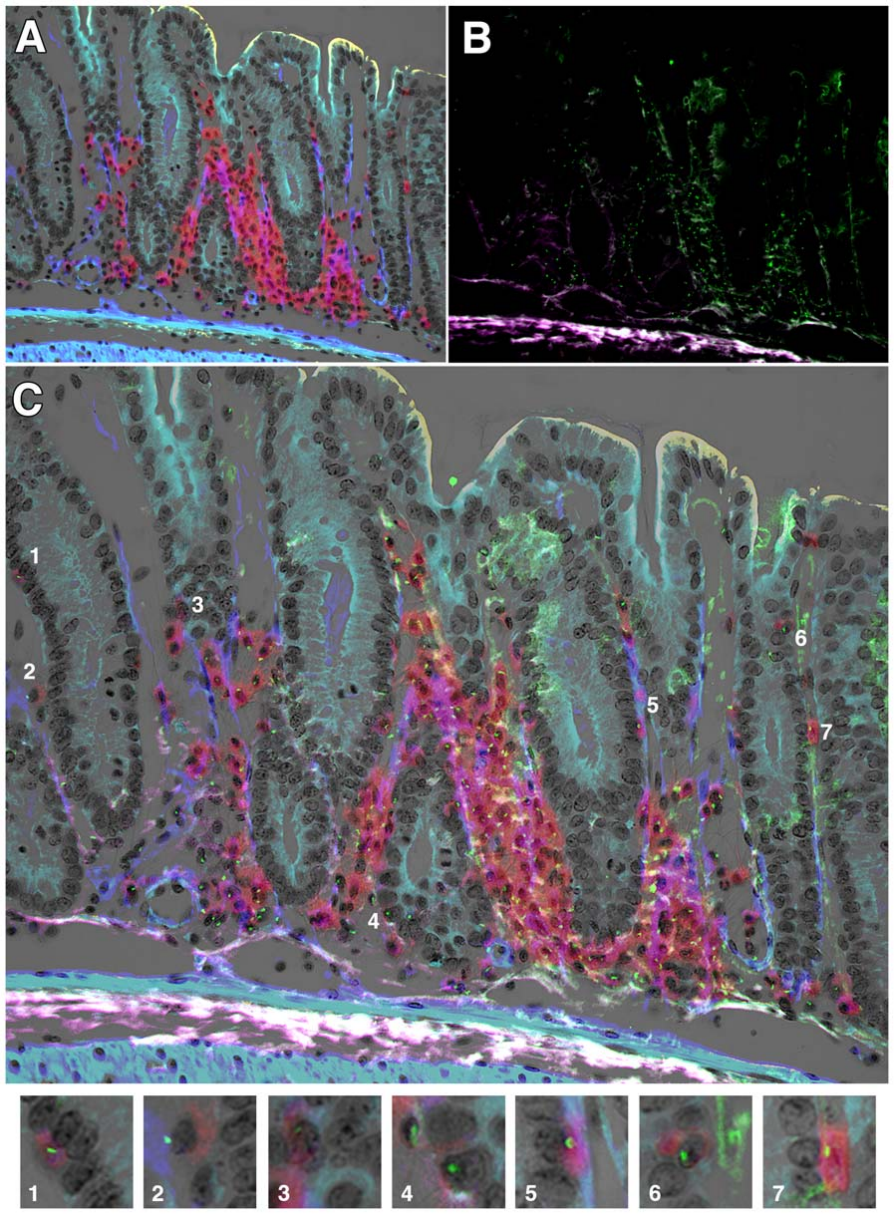
Double IHC with Y chromosome analysis. A. Double IHC for α-SMA (blue) and CD45 (red) in the colon of a female mouse with GvHD-like syndrome 28 days after male BMTx. Brightfield image (greyscale) overlain with fluorescent blue and red signals. B. Subsequent analysis of Y chromosome signals (green dots) on same section as A, showing some autofluorescence as pale green and white/purple. C. Overlay image of A and B with cells of interest highlighted and shown in Panels 1–7 below. Original magnifications A, B digitally reduced from 200X, C 200X. Panels further enlarged digitally to 600X.

We added an α-SMA IHC signal to sections of colon from female mouse with GvHD-like syndrome. In these analyses we stained the CD45 with Vector Red and α-SMA was detected using a Cy5-labelled second layer antibody which was pseudo-coloured blue to improve the visual contrast (Fig 4A). This is a composite image in which the brightfield greyscale image shows the tissue morphology, with light haematoxylin stained nuclei, and overlaying this image is a fluorescent one taken at the same time, showing CD45-positive cells in red and α-SMA-positive cells in blue. Some autofluorescence reveals the upper surface of the epithelium (yellow). The section was then reprobed for Y chromosomes (Fig 4B, green dots) and this image was combined with 4A as a composite (Fig 4C). Areas of interest are numbered and shown at a higher magnification in the panels below (1–7). An apparently male epithelial cell in the left hand crypt of Fig 4C was revealed to be CD45-positive (Panel 1). A stromal CD45-positive male cell is clearly shown in Panel 2. Panel 3 shows an oblique cut through a crypt edge with what resembles an epithelial cell being revealed as CD45-positive, as is the case in Panel 4. Panel 5 shows another apparently male epithelial cell decorated by the CD45 signal. Panel 6 shows another slightly oblique cut through the upper part of a crypt in which the lineage of the male cell is unclear until revealed to be CD45-positive. In Panel 7, another clearly Y chromosome-positive cell that could be epithelial, is again shown to be CD45-positive.

**Figure 5:**
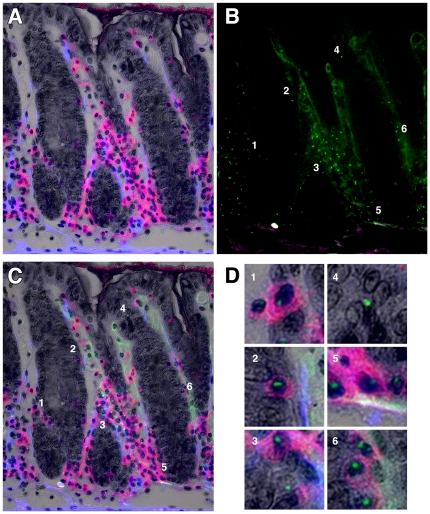
Triple IHC with Y chromosome analysis. A. Triple IHC for α-SMA (blue), CD45 (red) and *Dolichos biflorus* agglutinin (dark grey to black) in the colon of a female mouse with GvHD-like syndrome 28 days after male BMTx. Brightfield image (greyscale, nuclei counterstained with haematoxylin) overlain with fluorescent blue and red signals. B. Subsequent analysis of Y chromosome signals (green dots) on same section as A, showing some autofluorescence as pale green and purple. C. Overlay image of A and B, with cells of interest highlighted and shown in D Panels 1–6. Original magnifications A–C 200X. Panels in D are further digital magnifications of C to 1000X.

We stained a section parallel to that shown in Figure 4 additionally with direct *Dolichos biflorus* agglutinin epithelial staining as revealed by DAB (Fig 5A). This image has the same colours as shown in Figure 4, but the lectin has been coloured dark grey to black. Subsequent Y chromosome analysis showed many male cells in the section (Fig 5B). The overlay image (Fig 5C) shows several cells of interest (numbered 1–6 for clarity in both B and C) which are shown at higher magnification in the Panels in Fig 5D. In Panel 1, the male cell in the obliquely sectioned part of a crypt was shown to be CD45-positive, as were the apparently epithelial cells in Panels 2 and 3. Panel 4 shows a male cell that could be considered epithelial based upon the proximity of some darker stain of the DBA lectin and it not being CD45-positive, but on close inspection there are other nuclei to which this signal could be attributed, so its exact phenotype was not certain. This cell phenotype was rare and is shown for interest: all other examples of apparently male epithelial cells were also found to be CD45-positive. The male cells in Panels 5 and 6 were clearly CD45-positive.

### Y Chromosome FISH

The FITC-labelled Y chromosome paint gave reliable data throughout the study, with minimal batch-to-batch variability. Male control tissues from both SWR and Rag2 KO mice were equivalent (not shown). Female Rag2 KO mice given male BMTx displayed bone marrow-derived cells in several gastrointestinal compartments, which were maximal in Peyer's patches, and there were typical Y chromosome signals at the nuclear periphery (Fig 2D, 3C, 4C, 5C), whereas female tissues were negative. The Aqua and FITC fluorescent channels displayed tissue autofluorescence and were useful morphological guides in such images. There were differences in the maximum scores (days 28–38 post-BMTx) of Y chromosome-positive cells between tissues of the gut: lymphoid aggregates and single lymphoid cells within the stroma or epithelium produced a higher percentage of positive cells (70–80%) than either the stromal cells in general (∼60%) or the pericryptal myofibroblasts (10–30%) (Table 1). The reasons for these differences could include the nuclear:cytoplasmic ratio of the cells, as well as histological artefact due to section thickness, both of which would augment the signals from smaller cells with a high proportion of nuclear material. The proteolytic digestion time could also produce differential *in situ* hybridisation signals across a single section, which depend on chromatin structure in different cell types and their phenotype ([26] and Poulsom, unpublished observations). These results were comparable to control female Rag2 KO mice which had received a female BMTx (Table 2).

**Table 1:** Proportions of Y chromosome-positive cells in intestinal populations of female Rag2 KO mice receiving male SWR Bone Marrow.

	Days after BMTx				
	3	10	28	38	38
Small Intestine	*Control*	*Control*	*GvHD-like*	*DSS*	*Control*
Stroma	0±0 (4)	0.65±0.07 (3)	0.81±0.12 (10)	0.77±0.15 (5)	0.69 (1)
Lymphoid Aggregates	0±0 (4)	0.51±0.24 (4)	0.58±0.16 (3)	0.72±0.08 (3)	0.58 (1)
Pericryptal Myofibroblasts	0±0 (4)	0±0 (4) ***	0.03±0.06 (7) ***	0.10±0.14 (2) ***	0.20 (1)

	Days after BMTx				
	3	10	28	38	38
Colon	*Control*	*Control*	*GvHD-like*	*DSS*	*Control*
Stroma	0±0 (4)	0.62±0.07 (4)	0.74±0.12 (14)	0.76±0.06 (6)	0.73±0.04 (2)
Lymphoid Aggregates	0±0 (4)	0.51±0.14 (4)	0.71±0.15 (13)	0.60±0.16 (6)	0.74±0.16 (2)
Pericryptal Myofibroblasts	0±0 (4)	0.14±0.01 (4) ***	0.12±0.05 (14) ***	0.10±0.03 (6) ***	0.16±0.04 (2) **

Figures represent the mean % scores (± SD; (n) values) for either Y chromosome and CD45 double-positive cells in stroma or lymphoid aggregates, or Y chromosome and α-SMA double-positive PCF cells in the intestines of female Rag2 KO mice receiving male SWR BMTx on day 0. Stroma includes cells between 20 µm from the crypt basal edge and the *muscularis mucosae*. Pericryptal myofibroblasts were those α-SMA -positive cells inside 20 µm of the crypt basement membrane. No Y-positive epithelioid cells were found which were not also CD45-positive [ie of lymphoid origin]; those cells are included within the stromal counts. Only unequivocally double-labelled cells were scored, and so these counts represent an underestimate of the true values. No corrections have been made for section thickness (5 µm). Differences from Stroma group in the same tissue:

** p<0.01,

*** p<0.001 (Tukey-Kramer multiple comparisons post-test).

**Table 2:** Proportions of Y chromosome-positive cells in intestines of female Rag2 KO mice receiving male Rag2 KO Bone Marrow and of aged-matched untreated male control Rag2 KO and SWR mice.

	Male Rag2 KO into female Rag2 KO			Male Control Mice	
**Small Intestine**	Days after BMTx			*Rag2 KO*	*SWR*
	3	10	28		
Stroma	0	0.33±0.01	0.49 (1)	0.39±0.02	0.54±0.14
Lymphoid Aggregates	0	0.36±0.30	0.35 (1)	0.71±0.15	0.86±0.07
Pericryptal Myofibroblasts	0	0	0	0.44±0.08	0.29±0.09
**Colon**	Days after BMTx			*Rag2 KO*	*SWR*
	3	10	28		
Stroma	0	0.35±0.06	0.64±0.11	0.49±0.07	0.60±0.20
Lymphoid Aggregates	0	0.26±0.0	0.45±0.01	0.74±0.13	0.86±0.07
Pericryptal Myofibroblasts	0	0.05±0.01	0.08±0.04	0.34±0.06	0.32±0.06

Figures represent the mean % scores (± SD) for either Y chromosome-, CD45- double positive cells in stroma or lymphoid aggregates, or Y chromosome- and α-SMA-positive PCF cells in the intestines. N = 2 for all groups. Scoring details as in Table 1.

### X Chromosome FISH

Experiments altering probe concentrations, pepsin digestion times, the use of the sodium thiosulphate pre-hybridisation step, hybridisation temperatures and stringency washes did not improve the X chromosome ISH reliability. When we combined Y with X FISH, chromosome signals were either Y, XY, X or XX, and we were unable to detect evidence of cell-cell fusion (ie XXXY, XXY or XXX nuclei) in any of the intestinal cell populations that we studied (Fig 3B).

### Pathology of DSS and GvHD-like groups

**Figure 6:**
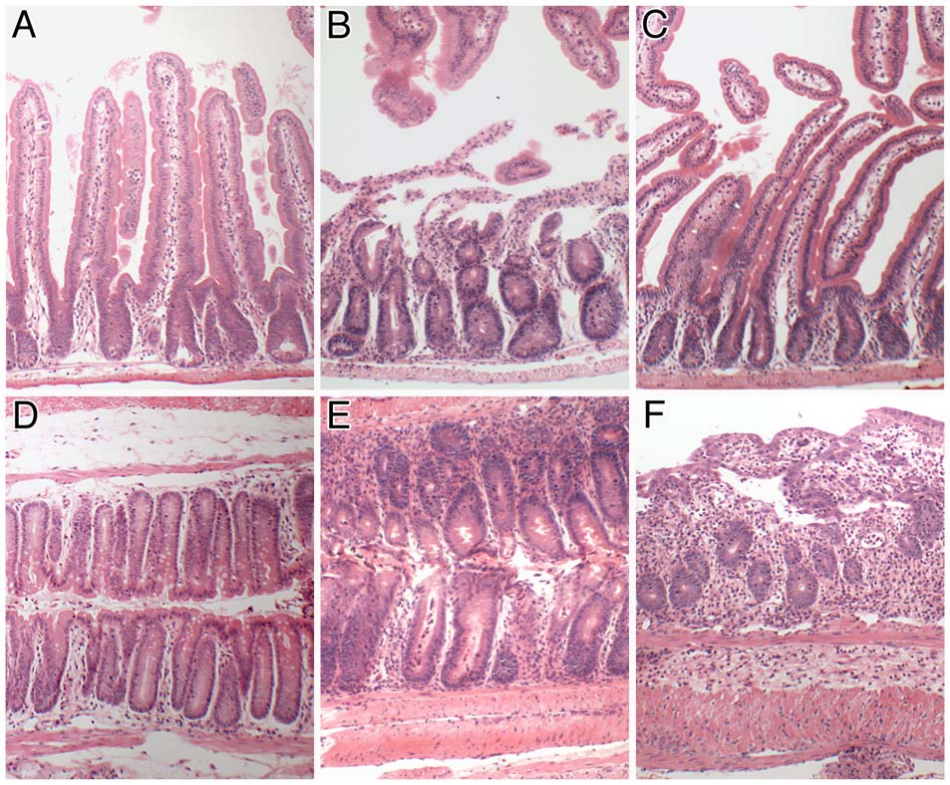
Histopathology of experimental mice. Small intestinal (A–C) and colonic (D–F) histology in female Rag2 KO recipients of male Rag2 KO BM 28 d previously (A, D), female Rag2 KO recipients of male SWR BM having a GvHD-like condition 28 d post BMTx (B, E) and female Rag2 KO mice in receipt of male SWR BM 28 d previously and given DSS for 5 days with 2 days recovery (C, F). Note normal histology in control mice (A, D), in contrast to small intestinal ulceration (B) and crypt abcesses (E) in GvHD-like mice, and crypt destruction and inflammation in colons (F) of DSS treated BMTx mice. Original magnifications 100X.

Haematoxylin and eosin stained sections of control small intestines (Fig 6 A–C) of female Rag2 KO recipients of male Rag2 KO BM (Fig 6A) and the small intestines of Rag2 KO female recipients of SWR male BM which had a GvHD-like condition (Fig 6B) and transplanted mice treated with DSS are shown (Fig 6C). The colon tissues of the same groups of mice are shown below (Fig 6D–F). Control Rag2 KO female mice given male Rag2 KO bone marrow showed no symptoms and their intestinal morphology was normal. Some of the sex-mismatched SWR BM-treated Rag2 KO mice displayed a GvHD-like syndrome, with weight loss, loose stools, fur ruffling and some hunching behaviour at 28 days post BMTx. Histological examination of their small intestinal tissues revealed inflammation with some loss of epithelial cells covering villi, and shortened crypts (Fig 6B). The small intestine of DSS-treated mice showed a normal to mild inflammatory response compared to control Rag2 KO mice (Fig 6C).

Colonic tissue in control transplanted Rag2 KO mice was normal (Fig 6D). Rag2 KO female mice receiving male SWR BM 28 d previously displayed a GvHD-like condition with crypts showing hyperplasia and crypt abcesses (Fig 6E). The DSS-treated Rag2 KO mice with SWR BM displayed large areas of ulcerated colon damage, with shortened and highly inflamed crypts (Fig 6F).

## Discussion

We have sought to clarify the identification of sex-mismatched allogeneic BMTx-engrafted cell phenotypes with host-derived cells by developing a method for the simultaneous detection of up to three histo- or immuno-histochemical signals with Y chromosome FISH on the same section. With nuclear or DNA counterstains and sequential capture of brightfield and up to 5 fluorescent filter channels we achieved up to 8 informative signals on a single histological section. We wanted to identify unequivocally the origin and functionality of epithelial and stromal cells in the intestines of lethally-irradiated mice receiving sex-mismatched BMTx rescue. We exploited the difference in lectin histochemistry between C57BL/6J and white Swiss Webster (SWR) mice with complementary epithelial and endothelial cell *Dolichos biflorus* agglutinin (DBA) binding [27].

By choosing DBA lectin staining to screen gut tissues, we hoped to identify crypts that had changed phenotype, and to analyse those for Y chromosomal and differentiation markers. In around 1.5×10^6^ crypts scored, all of the DBA lectin-negative cells we saw within the epithelial compartment existed as single cells which were also CD45-positive. Furthermore, all Y-positive cells within the epithelium were CD45-positive as well, even if they had an epithelioid morphology (Fig 2B). Such cells were morphologically so similar to the adjacent epithelium that they could be easily confused for such if not identified by another phenotypical marker of their origin. Thus we did not observe any unequivocal epithelial engraftment, either clonally throughout a crypt or as individual epithelial cells, of male donor cells into either the small or large intestinal epithelium at any time sampled to 38 days after BMTx. This is in agreement with our earlier studies of intestinal tissues after BMTx [9], [10], [12], [13], and other workers in this field [28]. In addition, combined XY chromosomal FISH did not reveal evidence of cell fusion events. We consistently observed donor-derived pericryptal myofibroblasts that appeared by 10 days and reached around 20% of those cells by 38 days. All these results suggests that normally a significant number of BM-derived myofibroblasts flux through the intestinal stroma.

The proportions of donor-derived lymphoid cells, as may be expected, were always greater than myofibroblasts in both the small and large intestines. It was of interest that there were no differences between the engraftment of donor BM-derived lymphoid or stromal CD45-positive cells in the control, DSS or GvHD-like groups of animals between days 28 and 38 post BMTx. This implies that the level of inflammation, tissue damage and repair had little impact on the overall flux of BM cells into these compartments. They all seemed to plateau at around 70% of Y-positive cells scored, which represents the level obtained by total BM replacement of haemopoietic stem cells [13]. A recent report suggests that proteinase-activated receptor 2 (PAR2)-expressing BM cells may be important in such homing in TNBS, but not DSS, induced colitis [28].

In contrast to the stromal engraftment of BM-derived cells, we saw very little into blood vessels in these experiments. The reasons for this are unclear. Prior work suggested that in TNBS colitis and in the stroma of an insulinoma model of cancer, neovasculogenesis and angiogenesis occurred with a significant stromal component being BM-derived [10], [29]. Those experiments used syngeneic mice with sex-mismatched BMTx with which to trace the Y-positive cells, in contrast to the allogeneic BM cells in our cross-strain chimaeras. In addition, there may be a lower inherent ability of endothelial cell engraftment in the Rag2 KO mice. This is supported by recent observations that immunodeficient SCID and Rag2 KO, as well as immunoglobulin-deficient Jh knockout mice, mobilised fewer haematopoietic stem cells (HSC) in response to G-CSF stimulus than zymosan [30]. If endothelial progenitor cells (EPCs) are mobilised to a similar extent to HSC, then the Rag2 KO hosts may not provide as permissive a milieu for the engraftment of SWR donor EPC into intestinal blood vessel endothelium.

Our data contrast with those from experiments using mice with *H felis*-infections which developed chronic inflammation leading to metaplasia, dysplasia and ultimately gastric carcinoma [25], in which BM-derived epithelial cells were seen after 20 weeks of infection, without cell fusion events. It may be that cells from infused bone marrow have differential abilities to home to different epithelia, and may be more likely in chronically inflamed tissues. Our data were obtained from a model of several acute episodes of colonic inflammation, and it is possible that not enough time elapsed to allow for measurable ingress of bone marrow-derived cells to the epithelium. Longer term experiments are needed to address this point. There may also be mouse strain differences which should be taken into account. Such bone marrow stem cell plasticity has been well reviewed recently [31]. Gastric epithelial cells have also been shown to fuse with human MSCs when the cells are co-cultured, but the MSCs failed to acquire an epithelial phenotype when cell contacts were prevented by a cell culture insert [32].

We were interested to compare our results with those of Rivzi and colleagues [33], who infused male recipient WT C57BL/6J or *Min* mice after 12 Gy irradiation with sex-mismatched GFP or Rosa26 bone marrow-derived cells. These authors reported widespread cell fusion in the small intestine of both recipient genotypes from as early as 2 weeks after irradiation and for up to 14 months after transplant in both normal villi and tumours, and occasionally in intestinal crypts. Cells of all four main small intestinal phenotypes were described to be both Y chromosome and GFP or β-gal-positive, with many patches of positive villous cells. The conclusion was that long-lived intestinal progenitors or stem cells were fusing with the BM-derived donor cells, but curiously only 1 in 400 crypts had β-gal-expressing cells though one might have expected there to be many crypts with fused cells, since the proportion of positive villi depicted seemed to be around 50%. It would be interesting to see the numbers of engrafting female cells in other compartments, as we would predict, and report here, that many of the pericryptal myofibroblasts would be donor-derived, and not fusion products. We were surprised that those authors found no CD45-positive cells within the epithelial compartment, as we saw these often. Those cells needed careful examination since on occasion their morphology resembled that of epithelial cells.

A recent publication [15] reported using female GFP-expressing whole bone marrow that was transplanted into irradiated (12 Gy) male wild-type and male IL-10^-/-^ colitic mice. These authors reported that patches of GFP-posi-tive gut epithelial cells could be seen which had either a Y chromosome or which possessed β-gal activity, both recipient traits. There was a high incidence of such events, and this could be reduced by treating the inflammation in IL-10^−/−^ colitic mice with 5-aminosalycilic acid (5-ASA). These fusion cells formed quite large contiguous areas, including the majority of cells in some intestinal crypts, and areas on villi. Between 1–7 days after transplantation, wild-type recipients of GFP^+^ whole bone marrow cells had at least one fusion cell at the crypt:villus junction in 37% of distal small intestinal crypts, while 21% of colonic crypts had at least one fusion cell at the mouth of the crypt: these events were considerably more common than previously reported by the group in the same experimental scenario [33]. They further showed that parabiosis of GFP and ROSA26 (β-gal) mice resulted in similar phenotypes when one partner was treated with DSS after surgical separation, and again that these phenomena were reduced by treatment by 5-ASA. These results concur with previous reports of BM-derived cell fusion in intestinal tumours [33], Purkinje neurons and haematopoietic or lymphocytic cells after inflammation or irradiation [34], [35]. We have some difficulty with the use of GFP as a marker of cell lineages, since it is not uniformly expressed in all cells, its expression may be exported by exosomes [36]–[38], and its fluorescence is similar to the often marked background autofluorescence of formalin-fixed tissues. It is a challenge to account for all these possibilities and be certain about cell fusion.

Another report recently showed apparently extensive engraftment of male donor MSCs (adherent cells) and HSCs (non-adherent cells) with female host intestinal epithelium in rats suffering TNBS-induced colitis [16]. Cultured HSCs, MSCs or combinations of both were prelabelled with BrdU and these populations were intravenously transplanted into non-irradiated rats one day after colitis induction. Gut tissues were found to contain considerable numbers of BrdU-labelled epithelial nuclei, often in whole crypts up to 21 days after the induction of colitis. The tissues were also probed for Y chromosome *Sry* sequences, which apparently corroborated the BrdU findings, but Y chromosome/BrdU-double positive cells were not shown. BrdU-positive cells were very rare without TNBS treatment.

These two experiments from mice and rats on crypt-wide engraftment and fusion of BM cells are perplexing for several reasons. Little prior evidence exists to indicate whole crypt engraftment in the gut. Intestinal crypts are clonal and are populated by stem cells near or at the base that produce all the cell types therein [39]–[41]. By cell division the stem cells and their transit-amplifying daughters provide a moving stream of cells which differentiate as they progress, and lose their ability to divide [but not to retain label] around half-way up the crypt [42]. Thus mature cells nearer the mucosal surface are derived from those lower down the cypts. To explain the BrdU staining of many adjacent crypts, as seen by Wei and colleagues [16], perhaps many successful engrafting events occurred from the infused BM-derived cells directly into the stem cell regions of many crypt bases, since their data show positive BrdU nuclei at this position. Alternatively, one successful stem cell niche engraftment led to that crypt dividing by fission many times to create a patch. However, they also showed uniformly intense BrdU staining of epithelial nuclei further up the same crypt. If these BrdU-positive donor cells were acting as normal stem cells, then there would be a decreasing gradient of nuclear BrdU staining intensity up the crypt, as the label in the lowest cells was diluted by 50% at each cell division. No such gradient was detectable in the data presented by Wei et al [16]. This leads one to wonder whether there could have been multiple simultaneous crypt epithelial engraftments (or fusions) all at the single cell level within multiple crypts, in addition to engraftments as several cell types, and so obtain uniform BrdU labelling of whole crypts. Such a mechanism seems unlikely to us. Human data from the Watanabe group (male bone marrow transplants (BMTx) into female patients) suggested that mostly single male cells engrafted into intestinal crypts [5], [6]. These workers did not detect either whole crypts or fusion events in their patients. This leaves the possibilities that the rat is substantially different from humans *vis à vis* its ability to engraft its intestines with BM-derived epithelial cells, or that there may have been considerable reutilisation of the BrdU that the infused cells contained. There is good evidence that this can occur in the neural tissues of mice and rats infused with tritiated thymidine or its analogues BrdU and CldU [43]. The use of genetically-labelled donor cells (β-gal or GFP) showed little or no engraftment, yet there was considerable BrdU or CldU labelling of nuclei when prelabelled similar cells were infused. In addition, uptake and nuclear labelling of neural tissues occurred even when dead cells or BrdU-labelled fibroblasts were infused. These results indicated that a substantial reutilisation of the nuclear label can take place, and that prelabelling of cells is not easy to interpret later on.

It is possible that there may be three paradigms for the engraftment of bone marrow cells into the epithelial compartment of the inflamed gut: (i) by cell ingress into the stem cell compartment, (ii) by occasional solo but non-stem cell events, possibly into the transit-amplifying compartment (and/or as single cell events into any of the intestinal cell phenotypes), or (iii) by fusion of BM-derived cells with cells in either of the preceding models. Cells may also fail to engraft at all. In this respect our recipient mice may have had residual radioresistant NK cells that might specifically eliminate bone marrow cells with epithelial potential. However, we find no evidence in the literature that points to such a possibility and we have noted a study by Tolar et al [44] that transplanted C57BL/6J multipotent adult progenitor cells (MAPC) into both Rag2^−/−^ and Rag2^−/−^/IL-2rγ_c_^−/−^ mice, and while they observed higher levels of MAPC engraftment in the tissues of Rag2^−/−^ mice, such a difference could be eliminated by prior 8Gy total body irradiation of the recipients. This suggests 8Gy is sufficient to eliminate any resistance to engraftment by NK cells, and pertinently our study irradiated the mice with 10Gy irradiation suggesting that residual NK cells would not be a confounding factor. We note that the radiosensitivity of NK cells has been reported to be significant; Rana et al found that *in vitro* gamma-irradiated human NK cells suffered massive cell death at doses above 6Gy, above which the authors were unable to measure any cytolytic activity against target K562 leukemic cells, even though there was a clear correlation between these parameters at lower doses [45]. Indeed, it has been demonstrated in PVG strain rats that a partial NK tolerance after 10Gy can be induced by radioresistant host cells receiving an MHC-mismatched BM transplant. Thus the *in vitro* alloreactivity of host Ly49i2 NK cells was dampened in the allogeneic chimeras, and not reversible by culture with IL-2 [46]. The trafficking of transplanted NK cells in mice has also been reported [47]. These authors gave 8 or 9 Gy to either Balb/c, FVB/N or C57BL/6 strains of mice followed by syngeneic or allogeneic T cell-depleted BM supplemented with purified donor NK cells. Concentrations of luciferase-positive NK cells were followed *in vivo* by a bioluminescent imaging method. They showed that L-selectin (CD26 ligand)-mediated allogeneic NK homing took place to lymphoid and GvHD target organs (i.e. skin and intestines), but to a lesser extent than with syngeneic NK cells. The gut NK homing integrin α4β7 was up-regulated after both syngeneic and allogeneic BMT to similar extents (18% and 25% of NK cells respectively). Despite the homing of NK cells to, and proliferation within, target organs, the allogeneic NK cells did not induce GvHD, even in unirradiated Rag2^−/−^ γ-chain^−/−^ recipients [47]. Overall, observations such as these suggest that the survival of host NK cells after irradiation was likely to be at a very low level, and unlikely to have much effect on the ingress and possible differentiation of BM-derived allogeneic cells into other cells types in receiving organs. This was clearly the case for the large numbers of Y-chromosome-positive allogeneic myofibroblasts in the colons of our experimental mice, with or without a GvHD-like condition. We feel confident to conclude that the lack of colonic epithelial cell engraftment in these experiments was not because of host attack on such donor-derived progenitors, but rather it reflected the exceedingly low probability that any of those cells took up residence in that specific compartment and made the appropriate cell lineage switch to become epithelial.

Which of the above paradigms is observed may depend on experimental or clinical circumstances and species differences. There is probably a spectrum of outcomes possible dependent on the cell biology of each system, the timing and frequency of observations and the degree to which it is possible to exclude false-positive or negative events. Whatever the mechanism(s) that explain these disparate results, we remain convinced that careful methods should identify each cell type unequivocally before a pronouncement is made on its identity.

## Materials and Methods

### Animals

All experiments were performed under strict UK Home Office regulations and a local Animal Ethics committee. All mice were young adults (4–6 weeks) and were housed in groups of 5 in filter-top cages under a 12∶12 hr light:dark cycle and given standard laboratory chow and drinking water *ad libitum*, in an enhanced social environment with nesting materials and play structures. Whole-body irradiation of mice (10 Gy) was delivered in two equal doses 3 hours apart using a ^137^Cs gamma source (IBL 637, CISBIO International/Schering Health Care, Burgess Hill, UK). Rag-2 mice on a C57BL/6J background were bred in-house. Swiss Webster (SWR) mice were purchased from Harlan (Bicester, UK). All mice received acidified drinking water for one week before irradiation to cleanse their gastrointestinal tracts of pathogens. Thirty one days after BM engraftment, DSS was added to their drinking water.

### Chemicals

Colitis was induced using DSS (MP Biomedicals, Eschwege, Germany; molecular weight 36–50,000 Daltons) administered in the drinking water (5% w/v) for 5 days followed by a 2 d recovery period [22]. *Dolichos biflorus* agglutinin-peroxidase (POD) conjugate (17 units per mg protein) was purchased from Sigma (Poole, Dorset, UK). On occasion an alkaline phosphatase (AP)-DBA conjugate was used from the same supplier. Vector Red and Vector Blue AP substrates were purchased from Vector Laboratories (Peterborough, UK), and used as recommended by the manufacturers. The antibodies used are shown in Table S1. X and Y-chromosome paints were purchased from Cambio Laboratories (Cambridge, UK) and were used as described by Fang et al (2005) [14] with slight modifications as described below. Other chemicals and solvents were of analytical grade purchased from BDH (Poole, Dorset, UK).

### BM harvest

Bone marrow was harvested from male SWR adult mice (6–8 weeks), which were killed by CO_2_ inhalation and cervical dislocation. Skin and muscle were removed from the hind leg long bones and tibiae and humeri were flushed through with 2 ml PBS using 27 gauge needles. Plugs of BM cells were “needled” without frothing through 19, 21 and 23 gauge needles in succession to dissociate clumps, and passed through a 40 µ cell strainer (Falcon, Becton-Dickinson, Oxford, UK). The cells were then spin-washed at 400 x *g* for 5 minutes, resuspended in PBS and nucleated cells were counted in a haemocytometer. A ratio of one donor mouse per 4 recipients was chosen, to provide 2×10^6^ nucleated cells that were injected slowly in 100 µl PBS through the warmed tail vein of the recipient mice within one hour of irradiation.

### Experimental designs

In pilot studies we used female SWR mice as recipients of male Rag-2 bone marrow cells. We reasoned that the DBA-negative epithelium of the small and large bowel of SWR mice would become lectin-positive if integration or fusion of BM cells occurred. We found that there was radiation-induced conversion of DBA-negative crypts into positive ones in both small and large intestine, and that none of these contained any Y chromosome-positivity in their epithelia (not shown). This phenomenon was probably related to the radiation-inducible mutation of the Dlb-1 locus seen in SWR mice [48]. Therefore we performed the reverse experiment for our main studies (Table 3), in which male SWR donor BM cells were injected into female Rag-2 mice, and these animals did not display a radiation-induced lectin conversion profile, as predicted by the results of Winton and colleagues for C57BL/6J-derived mice [48]. Control Rag-2 mice did show a small number of inherently DBA-negative crypts at the distal 1-2 cm of rectum, and so care was taken to exclude these areas in any analysis of possible BM engraftments.

**Table 3:** Experimental timelines.

Group	H^+^ H_2_O	BMTx	Tissue Harvest	Tissue Harvest	GvHD Harvest§	DSS 5d	Tissue Harvest
Male Rag2 to Female Rag2	−7	0	3	10			28
Male SWR to Female Rag2	−7	0	3	10	28	31	38
SWR Male Controls							*
Rag-2 Male Controls							*

“Rag2” denotes Rag2 knockout mice, SWR the Swiss Webster strain. Numbers represent days before or after BMTx (defined as time zero) for that event. BMTx (2×10^6^ nucleated BM cells in 100 µl PBS, *via* warmed tail vein) was performed within 1 hour of the second of two 5 Gy whole-body irradiations 3 hours apart. DSS was given as a 5% [w/v] addition to drinking water *ad libitum* for 5 days, with 2 days recovery. § Similar symptoms to graft versus host disease. * Age matched animals.

### Tissue harvesting and processing

The entire GI tract was removed, and divided into stomach, small and large intestines. The caeca were not used. The intestines were rinsed through with cold PBS, gently pressed flat and rolled in a proximal (inside) to distal (outside) manner and pinned as a “Swiss roll”, immersed in 20 volumes of methacarn fixative (methanol: chloroform: glacial acetic acid = 60∶30∶10) for 3 hours and stored in 70% ethanol at room temperature until routine paraffin wax embedding. In previous work [49], we found that methacarn provided cleaner lectin histochemistry than neutral-buffered formalin (NBF)-fixed tissues, and our IHC was unaffected by this. Other visceral organs were also harvested (liver, kidney, spleen) as well as skin. These latter organs were immersed in 20 volumes of 4% NBF for 24 hr, with one change after 6 hr, transferred to 70% ethanol and embedded in paraffin wax. Serial sections of the small and large intestinal blocks were cut at 5 µm at several levels for histological analyses. Other organs were sampled at two levels only.

### Lectin histochemistry

Sections were brought to water in a series of solvents (xylene, 100%, 95%, 70% ethanol). Endogenous peroxidase was blocked by incubating in 1.5% (w/v) H_2_O_2_ in methanol for 10 minutes. Endogenous alkaline phosphatase (AP) was blocked by immersing sections in 20% acetic acid in methanol for 15 minutes, followed by several PBS rinses. Sections were incubated in 3% BSA in PBS for 60 min to inhibit non-specific binding, followed by horseradish peroxidase- or AP-conjugated DBA lectin for 60 min. Three washes in PBS for 10 minutes were followed by addition of substrate as appropriate for the enzyme: either DAB (Sigma, Poole, UK) for peroxidase or Vector Blue or Vector Red for AP. Sections were routinely counterstained using Harris' haematoxylin, unless colour-contrast between combined brightfield and fluorescence overlay microscopy dictated otherwise (see Figure legends for details).

### Immunohistochemistry (IHC)

In general, 3-layer IHC was performed using an appropriate biotinylated second layer antibody and either streptavidin-peroxidase or streptavidin-alkaline phosphatase third layers (both from Dako, Glostrup, Denmark). Substrates were used as above. Microscope slides were either coverslipped with DPX (Sigma) or Vectashield Hard Set (Vector Labs; with or without DAPI as required), the latter giving faster release of the coverslips in warmed PBS, for subsequent FISH XY chromosome analyses (below).

Lymphoid cells were scored by IHC using antibodies to murine CD45 (leukocyte common antigen) followed by a fluorescent goat anti-rat IgG-Alexa-488 second layer (Molecular Probes, Oregon, USA), or by 3-layer IHC using the rabbit ant-rat Ig-biotin bridging antibody followed by streptavidin-HRP and DAB, as above. Epithelial cell phenotype was considered to be positive when using DBA lectin histochemistry since this stains the whole intestinal epithelium in Rag-2 mice (C57BL/6J background), and this stain was more reliable than a pan-cytokeratin antibody which tended to be differentially positive along the crypt and/or villus axes. A simplified protocol for triple IHC followed by Y-FISH is given in the Protocol S1.

### Brightfield microscopy

Brightfield areas of interest were photographed prior to XY chromosome FISH using an Orca-ER black and white digital camera (Hamamatsu Photonics, Hamamatsu City, Japan) on an Olympus BX 61 microscope with oil-immersion 20, 40 and 60X objective lenses, which was fitted with a BioPoint 2 motorised stage (Ludl Electronics Products, Hawthorne, NY, USA). Images were saved onto an Apple Macintosh G5 desktop computer running SmartCapture X3.02 (Digital Scientific, Cambridge, UK). At this stage fluorescent staining was also recorded using the 6-position filter wheel containing these epifluorescence channels: DAPI, Aqua, FITC, Cy3 and Cy5, as well as a brightfield position for transmitted light. SmartCapture preserves all the data from each channel separately for further individual or combined use, and the image panels for each of the fluorescent filter sets are precisely aligned. The Aqua channel gave a useful autofluorescence image which helped when overlaying subsequent FISH images using the SmartCapture filmstrip “Go” facility to reposition the field of view. This ensured that the same field of view was imaged for subsequent FISH analyses of those areas.

Vector Blue AP substrate is soluble in xylene and so sections could not be mounted in DPX. In these cases a water-based mountant such as Glycergel (Dako) or VectorShield HardSet was used. On occasion we detected CD45 using 3-layer IHC to AP-conjugates with Vector Red substrate, or 2-layer IHC to Alexa-488 conjugated goat anti-rat IgG.

### Fluorescent in situ hyridisation for Y and X chromosomes

We modified our published method [11] by using sections previously stained and photographed in brightfield (see above) for DBA lectin or other IHC signals, including any fluorescent IHC that may be digested away during pepsin treatments in subsequent FISH steps. In pilot XY FISH studies, we found that a 10 min 80°C sodium thiocyanate step was optimal for methacarn-fixed tissues to improve nuclear penetration of DNA probes. In addition, we found that for such tissues a pepsin digestion step of 2.5–3 minutes was sufficient for the intestines, somewhat shorter than that normally applied to routine NBF-crosslinked tissues (typically 10 minutes). After hybridisation and washes in 0.5X SSC at 37°C followed by several in PBS over 15 minutes, sections were mounted in Vectashield Hard Set and stored at 4°C in the dark. The X chromosome paint produced red signals inside nuclei, while the Y chromosome paint gave green signals, and their nuclear positions were consistent with their chromosomal addresses. A simplified protocol for Y-FISH is given in the Protocol S1.

### FISH Image analyses

Where indicated in the Figures, brightfield and first-round fluorescent images were obtained for IHC and/or lectin histochemistry as described above. The SmartCapture “Go” facility was used to rephotograph the same areas of interest from the first analyses. The Cy3 channel was used for X chromosomes and FITC channel for the Y, with the Aqua channel to assist the image overlay.

We observed that the short pepsin digestion times we used after initial IHC or lectin imaging caused minimal distortion of the FISH-probed sections. Even so, very small errors were possible, and so landmark strucures, such as CD45-positivity with DAB, which appeared densely black in the fluorescent image mode, and which was common to both images, were aligned before any further analyses were made. We present examples of such images and their overlay in Figure S1, and measurements of a series of images at the two stages are given in Table S2.

### Cell scoring

Brightfield DBA lectin (positive or negative) histochemistry was used as a primary screen for likely host-donor differences with areas of interest photographed along with the accompanying IHC when performed, using the appropriate filters on the microscope. Subsequent XY chromosome analyses were done in fluorescence mode, where DAPI-counterstained nuclei were assessed for positional and chromosomal features. A 30 sec haematoxylin nuclear stain was compatible with subsequent DAPI fluorescent staining, but stronger staining (2 minutes) blocked some of the DAPI fluorescence. In some analyses we used brightfield IHC images combined with the background fluorescence in the Aqua channel to obtain a hybrid overlay that showed nuclei and other structures well. This meant that we did not always need to use the DAPI channel (blue) for nuclei, and so we could falsely colour another fluorescent IHC image blue for the overlays. Combined brightfield and fluorescent overlay imaging was used to facilitate cell scoring. Images from SmartCapture were exported as TIFF images and then imported into Adobe Photoshop for overlay analyses, where opacity was the only digital manipulation, and in which the “upper” layer, containing fluorescent IHC and/or XY chromosomal images, was made 60% opaque, with no alteration in the lower layer.

We scored the brightfield lectin histochemistry at 100X overall magnification of all small and large intestinal crypts at between 25 and 45 levels of all the intestines of all mice (total of approximately 1,500,000 crypts). Serial sections of many of these levels were stained for CD45 BM-derived cells and α-smooth muscle actin (α-SMA) positivity, together with Y-chromosome analysis. We cannot report X-chromosome analysis on all sections since the batches of X paint gave inconsistent signals and a variable background. Even with these limitations, the analyses we performed did not produce any evidence of cell fusion events (XXX, XXY or XXXY per nucleus) in any of the intestinal or lymphoid tissues studied.

### Statistical analyses

Quantitative data were subjected to statistical analyses using GraphPad Prism (version 4.00 for Apple Macintosh, GraphPad Software, San Diego California USA; www.graphpad.com). A null hypothesis was rejected at a p value of less than 0.05.

## Supporting Information

Figure S1 **Alignment of twin brightfield and fluorescent images.** Colonic section from a Rag2 KO mouse that received SWR BMTx 38 days previously, and 5% DSS in the drinking water for 5 days from day 31 with 2 days' recovery. A. Combined brightfield and fluorescent image with CD45 cells stained black, DBA lectin in red and haematoxylin counterstained nuclei, as well as α-smooth muscle actin stained myofibroblasts revealed using Cy5 conjugated secondary antibody, falsely coloured blue. Note lines 1 and 2 transposed from image B, marking the anchor points from that image used subsequently to align the overlay of the two images, and to illustrate any linear alteration after digestion for Y-FISH. Note a lectin negative crypt (a), a partially DBA-positive crypt (b), a small vascular vessel (c) and a wholly DBA-positive crypt (d). Arrowheads denote positions of Y signals in crypt epithelial cells seen in B. B. Fluorescent image of the same section in A that has been pepsin digested and probed for Y chromosomes (green dots), which show clearly against the autofluorescent background. Aqua channel autofluorescence has been falsely coloured magenta to contrast that of the green signals. Structures labelled as in Fig 1A. Note the white strip along the right hand edge, which represents the error in the motorised microscope stage in returning to the position of the image taken in A. C. Overlay image of Fig 1A and 1B. In Adobe Photoshop Fig 1A was a layer “underneath” Fig 1B, with the latter being adjusted so that its opacity was set to 50% to allow both images to be seen. Structures labelled as in A and B. Note the Y-positive cells highlighted by the arrowheads which could be interpreted as epithelial were they not also found to be CD45-positive. The negative crypt (a) was predicted to be Y positive by our central hypothesis, but was found to contain no Y chromosome signals, as was found for all the other crypts. All Y positive cells within the epithelial compartment were also found to be CD45-positive, and so of lymphoid origin. The vascular channel (c) also contained such a cell, even though it may have been considered endothelial at first glance. The lines 1 and 2 demonstrate that positional errors caused by distortion following pepsin treatment of the section for Y-FISH were minimal, and were consistent with CD45 positively stained cells.
(TIF)

Table S1 **DBA Lectin stains Rag-2 gut epithelium and SWR blood vessel endothelium.** CD45 (leukocyte common antigen, LCA) stains all lymphoid lineages from BM. α-smooth muscle actin stains pericryptal or other myofibroblasts, plus smooth muscle cells of blood vessels and the outer gut wall. ‡ 30% Hydrogen peroxide (BDH) was added at a dilution of 1∶500. * Manufacturer's instructions were followed: 2 drops each of reagents A, B & C in 5 ml Tris-HCl pH 8.5.
(DOC)

Table S2 **The table shows the on-screen measurements (centimetres) between 2 points on paired Photoshop tiff images taken before and after FISH, and their ratios.** Slides were taken from all experimental groups. The measurements show a very slight shrinkage after the FISH protocol. The points were taken from 3 DAB-stained CD45-positive cells per image, and which were approximately at right angles to each other, in order to estimate the distortion in the X and Y axes. These cells could be easily identified in both images before and after FISH. These changes were not found to alter the assigning of the Y chromosome nuclear signals to the overlay image from the previous IHC staining. In all cases, the alignment of images was performed before the Y signal channel was turned on, to obviate any prejudicial placement. Mean ratio ± SD (n = 20) 0.98±0.02.
(DOC)

Protocol S1 **Details of the triple immunohistochemistry and subsequent chromosomal** ***in situ*** **hybridisation methods may be found in the Protocol S1 online.**
(DOC)
