# Supplementary figures and images for: Bone Marrow Cells in Murine Colitis: Multi-Signal Analysis Confirms Pericryptal Myofibroblast Engraftment without Epithelial Involvement
Source: PLoS One. 2011 Oct 13;6(10):e26082. doi: 10.1371/journal.pone.0026082 (PMC3192776; doi:10.1371/journal.pone.0026082)

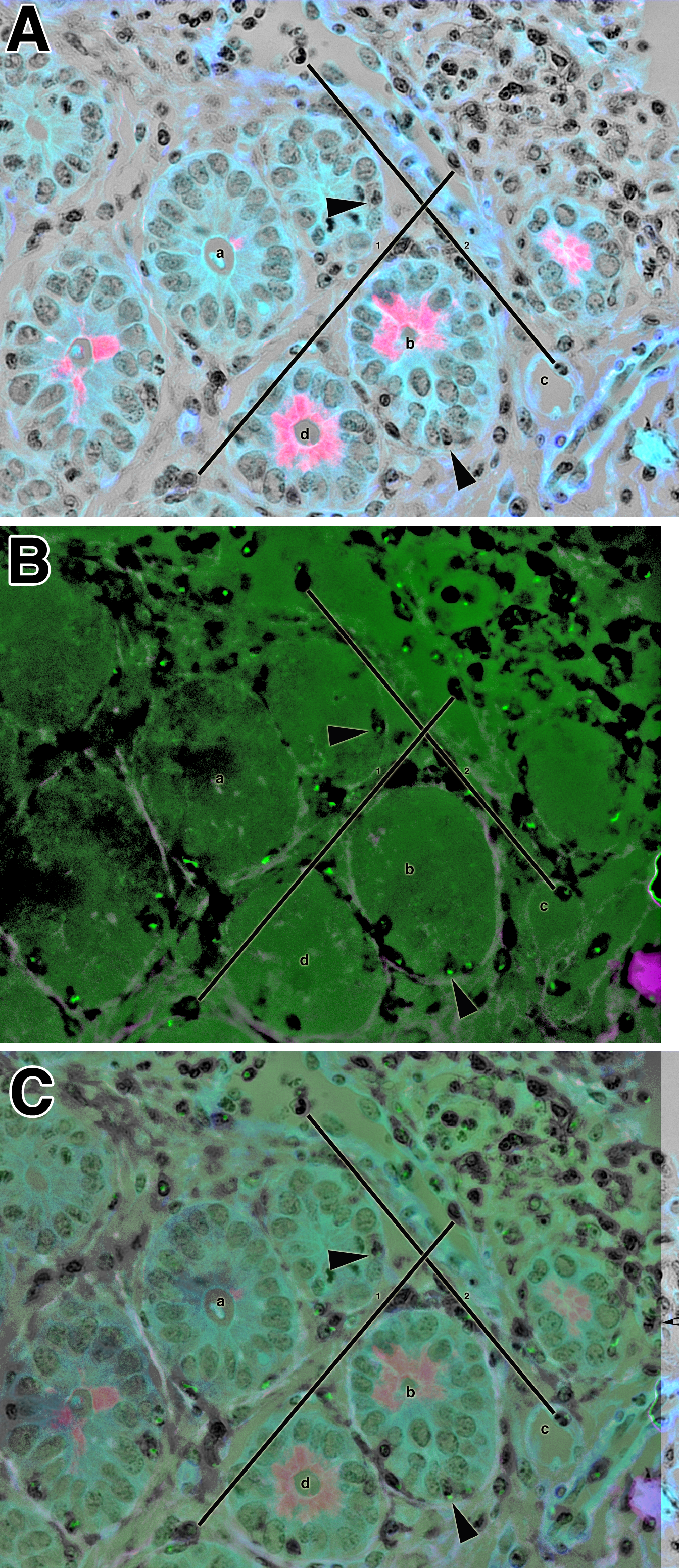

Supplement: Figure S1 — Alignment of twin brightfield and fluorescent images. Colonic section from a Rag2 KO mouse that received SWR BMTx 38 days previously, and 5% DSS in the drinking water for 5 days from day 31 with 2 days' recovery. A. Combined brightfield and fluorescent image with CD45 cells stained black, DBA lectin in red and haematoxylin counterstained nuclei, as well as α-smooth muscle actin stained myofibroblasts revealed using Cy5 conjugated secondary antibody, falsely coloured blue. Note lines 1 and 2 transposed from image B, marking the anchor points from that image used subsequently to align the overlay of the two images, and to illustrate any linear alteration after digestion for Y-FISH. Note a lectin negative crypt (a), a partially DBA-positive crypt (b), a small vascular vessel (c) and a wholly DBA-positive crypt (d). Arrowheads denote positions of Y signals in crypt epithelial cells seen in B. B. Fluorescent image of the same section in A that has been pepsin digested and probed for Y chromosomes (green dots), which show clearly against the autofluorescent background. Aqua channel autofluorescence has been falsely coloured magenta to contrast that of the green signals. Structures labelled as in Fig 1A. Note the white strip along the right hand edge, which represents the error in the motorised microscope stage in returning to the position of the image taken in A. C. Overlay image of Fig 1A and 1B. In Adobe Photoshop Fig 1A was a layer “underneath” Fig 1B, with the latter being adjusted so that its opacity was set to 50% to allow both images to be seen. Structures labelled as in A and B. Note the Y-positive cells highlighted by the arrowheads which could be interpreted as epithelial were they not also found to be CD45-positive. The negative crypt (a) was predicted to be Y positive by our central hypothesis, but was found to contain no Y chromosome signals, as was found for all the other crypts. All Y positive cells within the epithelial compartment were also found to be CD [file pone.0026082.s001.tif]
