# Supplementary material for: Bone Marrow Cells in Murine Colitis: Multi-Signal Analysis Confirms Pericryptal Myofibroblast Engraftment without Epithelial Involvement
Source: PLoS One. 2011 Oct 13;6(10):e26082. doi: 10.1371/journal.pone.0026082 (PMC3192776; doi:10.1371/journal.pone.0026082)
Supplement: Table S1 — DBA Lectin stains Rag-2 gut epithelium and SWR blood vessel endothelium. CD45 (leukocyte common antigen, LCA) stains all lymphoid lineages from BM. α-smooth muscle actin stains pericryptal or other myofibroblasts, plus smooth muscle cells of blood vessels and the outer gut wall. ‡ 30% Hydrogen peroxide (BDH) was added at a dilution of 1∶500. * Manufacturer's instructions were followed: 2 drops each of reagents A, B & C in 5 ml Tris-HCl pH 8.5. (DOC) [file pone.0026082.s002.doc]

**Supporting Information**

**Table S1**. **Lectin and IHC reagents, X & Y chromosome FISH reagents**

| **Reagent** | **Supplier** | **Dilution** |
| --- | --- | --- |
| DBA-POD-conjugated lectin | Sigma | 1:100 |
| DBA-alkaline phosphatase-conjugated lectin | Sigma | 1:100 |
| Rat anti-mouse CD45 (purified monoclonal, IgG) | BD Pharmingen | 1:20 |
| Mouse anti-α-smooth muscle actin (monoclonal, IgG) | Sigma | 1:4000 |
| Rat anti-mouse endomucin (monoclonal, IgG) | Santa Cruz | 1:100 |
| Rabbit anti-rat Ig-biotin conjugate (2^nd^ layer) | Dako | 1:100 |
| Goat anti-rat IgG-Alexa-488 conjugate (detecting layer) | Molecular Probes | 1:200 |
| Goat anti-mouse IgG-Cy5 conjugate (detecting layer) | Zymed | 1:50 |
| Streptavidin-POD conjugate (detecting layer) | Dako | 1:500 |
| Streptavidin-AP conjugate (detecting layer) | Dako | 1:100 |
| 3,3’Di-amino benzidine (POD substrate)‡ | Dako | 0.5mg/ml |
| Vector Red (AP substrate) | Vector | * |
| Vector Blue (AP substrate) | Vector | * |
| X Chromosome paint-Cy3 conjugate | CamBio | 1:4 |
| Y Chromosome paint-FITC conjugate | CamBio | 1:4 |
