# Supplementary material for: Bone Marrow Cells in Murine Colitis: Multi-Signal Analysis Confirms Pericryptal Myofibroblast Engraftment without Epithelial Involvement
Source: PLoS One. 2011 Oct 13;6(10):e26082. doi: 10.1371/journal.pone.0026082 (PMC3192776; doi:10.1371/journal.pone.0026082)
Supplement: Table S2 — The table shows the on-screen measurements (centimetres) between 2 points on paired Photoshop tiff images taken before and after FISH, and their ratios. Slides were taken from all experimental groups. The measurements show a very slight shrinkage after the FISH protocol. The points were taken from 3 DAB-stained CD45-positive cells per image, and which were approximately at right angles to each other, in order to estimate the distortion in the X and Y axes. These cells could be easily identified in both images before and after FISH. These changes were not found to alter the assigning of the Y chromosome nuclear signals to the overlay image from the previous IHC staining. In all cases, the alignment of images was performed before the Y signal channel was turned on, to obviate any prejudicial placement. Mean ratio ± SD (n = 20) 0.98±0.02. (DOC) [file pone.0026082.s003.doc]

**Supporting Information**

**Table S2. Pre- and post-hybridisation tissue measurements on colonic sections.**

| **Before FISH** | **After FISH** | **Ratio** | **Before FISH** | **After FISH** | **Ratio** |
| --- | --- | --- | --- | --- | --- |
| 13.85 | 13.61 | *0.98* | 31.71 | 31.01 | *0.98* |
| 15.65 | 15.08 | *0.96* | 10.78 | 10.60 | *0.98* |
| 15.56 | 15.19 | *0.98* | 10.80 | 10.64 | *0.98* |
| 7.98 | 7.56 | *0.94* | 15.88 | 15.11 | *0.95* |
| 13.23 | 12.96 | *0.98* | 15.85 | 15.34 | *0.97* |
| 20.37 | 20.32 | *1.00* | 17.52 | 17.14 | *0.98* |
| 14.65 | 14.42 | *0.98* | 20.30 | 20.21 | *1.00* |
| 15.38 | 15.23 | *0.99* | 20.27 | 20.11 | *0.99* |
| 8.78 | 8.28 | *0.94* | 20.64 | 20.49 | *0.99* |
| 16.25 | 16.20 | *1.00* | 17.73 | 17.60 | *0.99* |
