# Supplementary material for: Bone Marrow Cells in Murine Colitis: Multi-Signal Analysis Confirms Pericryptal Myofibroblast Engraftment without Epithelial Involvement
Source: PLoS One. 2011 Oct 13;6(10):e26082. doi: 10.1371/journal.pone.0026082 (PMC3192776; doi:10.1371/journal.pone.0026082)
Supplement: Protocol S1 — Details of the triple immunohistochemistry and subsequent chromosomal in situ hybridisation methods may be found in the Protocol S1 online. (DOC) [file pone.0026082.s004.doc]

**Protocols**

***Protocol 1. Detection of CD45, DBA lectin & α-SMA by IHC & XY Chromosomes by FISH***

1. Paraffin wax sections (5µm) were dewaxed in xylene and brought to water through graded alcohols. Endogenous peroxidases were blocked by incubation in H_2_O_2_ (1:40 dilution of 30% stock solution) in methanol for 10 min. These steps were performed on a TissueTek Prisma 6132 processor (Sakura Finetek (Europe), Zoeterwoude, Netherlands).

2. Endogenous AP was blocked as needed by a separate manual incubation in 20% acetic acid in methanol for 15 min.

3. After rinses in tap water and PBS an antibody blocking step was carried out in 3% BSA in PBS for 30 min. All IHC steps were carried out in a humid chamber at room temperature.

4. All antibodies and lectins were diluted in 3% BSA in PBS and applied for 60 min. The first antibody to be applied was monoclonal rat anti-mouse CD45.

5. Extensive washing in PBS 4 times was carried out between all IHC layers over 15 min.

6. Second incubations were mouse anti-α-SMA mixed with rabbit anti-rat IgG-biotin, 60 min.

7. Washes in PBS, as step 5.

8. Third layers were streptavidin-peroxidase mixed with goat anti-mouse IgG-Cy5 and DBA-AP, 60 min.

9. Washes as step 5.

10. CD45 was developed after its streptavidin-POD third layer using DAB (0.5mg/ml plus 20µl of 30% H_2_O_2_ per 10ml reagent [1:500]) for 5 min, followed by extensive PBS washing.

11. Sections for DBA-AP development were washed in two changes of AP buffer (200mM Tris-HCl pH 8.5) for 5 min to equilibrate the pH and remove PBS. AP was developed using freshly prepared VectorRed (brightfield and fluorescent) or VectorBlue (brightfield only) substrates as per the manufacturer’s instructions (two drops each of reagents A, B and C into 5 ml of AP buffer) for either 2 min (fluorescent microscopy) or 5 min (brightfield microscopy). An additional 2 washes with AP buffer were performed after staining.

12. A light nuclear counterstain using Harris’ haematoxylin for 5 seconds was carried out, followed by tap water blueing, and subsequent dehydration through graded alcohols to xylene (TissueTek Prisma), and coverslipping under DPX, Glycergel or VectaShield Hard Set as appropriate.

***Protocol 2: FISH after triple staining***

1. IHC-stained sections in DPX were immersed in at least 3 changes of xylene over 48 hours to release the coverslips without distorting the sections. A further incubation for 12 hours in fresh xylene was performed to remove all traces of DPX. Where Vectashield was used as mountant, coverslips were removed in PBS at 37˚C for 30 minutes.

2. Sections were rehydrated to PBS on the tissue processor.

3. Nuclear permeabilisation was performed by incubating in fresh 1M sodium thiocyanate for 10 min at 80˚C. Two PBS washes of 5 min were performed.

4. Sections were treated with fresh 4 mg/ml pepsin in 0.1M HCl for 2.5-3 min at 37˚C.

5. Pepsin was quenched using 2 mg/ml glycine for 5 min at room temperature, followed by 2 washes in PBS for 5 min.

6. A post-fixation in fresh 4% PFA in PBS was performed for 2 min, and a wash in PBS.

7. Sections were dehydrated to absolute ethanol and air-dried at room temperature.

8. 15µl of Y chromosome paint, or both X and Y chromosome paints (1:4 in hybridisation buffer) was added to each section, coverslipped and sealed with vulcanising rubber solution (Weldtite Products, Barton on Humber, UK).

9. A denaturation step at 60˚C was carried out for 10 min before incubating overnight in a sealed humid chamber at 37˚C.

10. The rubber cement was removed with forceps, the coverslip simultaneously removed and washed in 0.5X SSC at 37˚C for 5 min.

11. Slides were washed extensively in PBS over 15 min and mounted in VectaShield Hard Set, with or without DAPI as required. All slides were archived at 4˚C in the dark, and were stable for at least 2 months.
